# Overexpression of a Maize Sulfite Oxidase Gene in Tobacco Enhances Tolerance to Sulfite Stress via Sulfite Oxidation and CAT-Mediated H_2_O_2_ Scavenging

**DOI:** 10.1371/journal.pone.0037383

**Published:** 2012-05-31

**Authors:** Zongliang Xia, Kaile Sun, Meiping Wang, Ke Wu, Hua Zhang, Jianyu Wu

**Affiliations:** 1 College of Life Science, Henan Agricultural University, Zhengzhou, People’s Republic of China; 2 Key Laboratory of Physiology, Ecology and Genetic Improvement of Food Crops in Henan Province, Zhengzhou, People’s Republic of China; Friedrich-Alexander-University Erlangen-Nurenberg, Germany

## Abstract

Sulfite oxidase (SO) plays an important role in sulfite metabolism. To date, the molecular mechanisms of sulfite metabolism in plants are largely unknown. Previously, a full-length cDNA of the putative sulfite oxidase gene from maize (*ZmSO*) was cloned, and its response to SO_2_/sulfite stress at the transcriptional level was characterized. In this study, the recombinant ZmSO protein was purified from *E.coli*. It exhibited sulfite-dependent activity and had strong affinity for the substrate sulfite. Over-expression (OE) of *ZmSO* in tobacco plants enhanced their tolerance to sulfite stress. The plants showed much less damage, less sulfite accumulation, but greater amounts of sulfate. This suggests that tolerance of transgenic plants to sulfite was enhanced by increasing SO expression levels. Interestingly, H_2_O_2_ accumulation levels by histochemical detection and quantitative determination in the OE plants were much less than those in the wild-type upon sulfite stress. Furthermore, reductions of catalase levels detected in the OE lines were considerably less than in the wild-type plants. This indicates that SO may play an important role in protecting CAT from inhibition by excess sulfite. Collectively, these data demonstrate that transgenic tobacco plants over-expressing *ZmSO* enhance tolerance to excess sulfite through sulfite oxidation and catalase-mediated hydrogen peroxide scavenging. This is the first *SO* gene from monocots to be functionally characterized.

## Introduction

As a molybdenum-containing enzyme, sulfite oxidase (SO; EC 1.8.3.1) catalyzes the oxidation of sulfite to sulfate, and thus plays important roles in diverse metabolic processes such as sulfur detoxification and purine catabolism [1], [2], [3].

SO has been identified from prokaryotes such as *Thiobacillus thioparus* [4], *Thiobacillus novellus* [5], [6], and *Thiobacillus acidophilus* [7], and from eukaryotes such as humans [8], [9], chicken [10], mouse [11], and *Arabidopsis thaliana* [12]. The well-known vertebrate SO is a homodimer and is localized within the mitochondria [8]. It contains three functional domains: an N-terminal heme domain, a molybdenum cofactor (MoCo) domain, and a C-terminal dimerization domain [13]. Several animal sulfite oxidases have been crystallized and their catalytic mechanisms have been unraveled [1], [13], [14]. Deficiency of the enzyme in humans leads to severe neurological abnormalities and early death [15].

In higher plants, *A. thaliana* SO (AtSO) was the first identified and biochemically characterized sulfite oxidase [12], [16], [17], [18]. Compared with animal SO, AtSO lacks the heme domain and possesses a molybdenum center alone and thus is the simplest Mo-enzyme [12], [16]. AtSO shows sulfite-dependent oxidizing activity with ferricyanide as an artificial electron acceptor [12]. Recent biochemical evidence has revealed that AtSO utilizes molecular oxygen as a natural electron acceptor, ultimately resulting in the formation of hydrogen peroxide [17], [18]. Studies on the physiological roles of plant SO have been lagging behind biochemical studies. Most reports have been made in recent years. The SO proteins from *A. thaliana* and *N. benthamiana* have been confirmed to be involved in sulfite/sulfur dioxide detoxification *in planta* by genetic approaches [19], [20], [21]. AtSO is localized in peroxisomes, but it co-regulates the sulfate assimilation pathway with the chloroplast-localized enzyme adenosine 5′-phosphosulfate reductase (APR) [22], [23].

In spite of the progress made in understanding molecular and biological function of SO in model plants, the knowledge of molecular and functional aspects of the SO proteins from higher plants is still limited. Maize (*Zea mays*) is an important cereal crop worldwide that is a staple food to many populations. Environmental pollutants such as sulfur dioxide/sulfite and acid rain adversely affect maize growth and development by inducing occurrence of physiological diseases, thus they are becoming serious problems in several maize-planting regions of Northern China [24].Unfortunately, molecular mechanisms underlying sulfite metabolism in plants are largely unknown, let alone in crop plants. In our recent work, a putative sulfite oxidase gene from maize (*ZmSO*) was cloned by RACE-PCR, and its response to SO_2_ stress was characterized [25]. However, the biochemical properties and physiological functions of the putative crop SO during sulfite stress are still unclear. Here we characterized the putative maize SO homolog in transgenic tobacco to investigate sulfite stress tolerance and possible detoxification mechanisms.

## Results

### Sulfite Oxidase Activity of ZmSO *in vitro*

**Figure 1:**
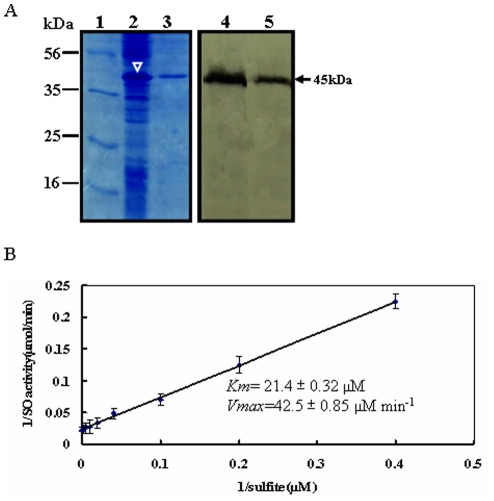
Expression, purification and kinetic analysis of recombinant maize sulfite oxidase. (A) Purification of histidine-tagged maize SO. The histidine-tagged ZmSO (with a predicted molecular mass of 45 kDa) was overexpressed in bacterial cells induced by isopropyl-β-D-thiogalactopyranosid (lane 2, indicated by white arrowhead). The overexpressed protein was purified using metal chelate affinity chromatography (lane 3). The overexpressed and purified SO proteins were confirmed by Western blot with an anti-histidine monoclonal antibody (lane 4 and lane 5; signals marked by an arrowhead). The molecular mass markers (kDa, lane 1) are shown on the left. (B) Steady-state kinetics of recombinant ZmSO with sulfite. Double-reciprocal presentation (Lineweaver-Burk plot) of enzyme rate was conducted using varying concentrations of sulfite (2.5, 5, 10, 25, 50, 100, 200, and 400 µM) and constant 400 µM ferricyanide. The reaction was initiated with 1.0 µg of purified recombinant ZmSO.

In our recent study, the full-length cDNA of *ZmSO* (accession number: FJ436404) was obtained by 5′- and 3′-RACE- PCR. The putative ZmSO exhibits strong similarity to other plant orthologs. Using *ZmSO* full length sequence as a query probe, an *in silico* searching has revealed that *ZmSO* is located on chromosome 1 in maize genome [25].To ascertain whether the isolated *SO* ortholog from maize encodes a functional sulfite oxidase, a hexahistidine-tagged ZmSO was expressed in bacterial cells and purified using nickel chelate affinity chromatography (Fig. 1A; left panel). The purified ZmSO proteins had a molecular mass of 45 kDa as shown by Western blot experiments using monoclonal antibodies against the hexahistidine-tag. This mass was in agreement with predictions. (Fig. 1A; right panel).

In biochemical assays, the recombinant ZmSO protein exhibited a sulfite-dependent activity when ferricyanide was used as an electron acceptor. The Michaelis constant (*K_m_*) value for sulfite in the ferricyanide assay was determined to be 21.4±0.32 µM (Fig. 1B). The maximum velocity (*V_max_*) value for sulfite was determined to be 42.5±0.85 µM min^−1^ by extrapolation from Lineweaver-Burk plots (Fig. 1B). Similar kinetic constants were obtained in several independent assays. For the recombinant ZmSO protein, the optimal pH for enzyme activity was found to be ∼pH 8.0 (data not shown). These results indicated that ZmSO has sulfite oxidase activity *in vitro*.

### Construction of *ZmSO* Transgenic Tobacco Lines

**Figure 2:**
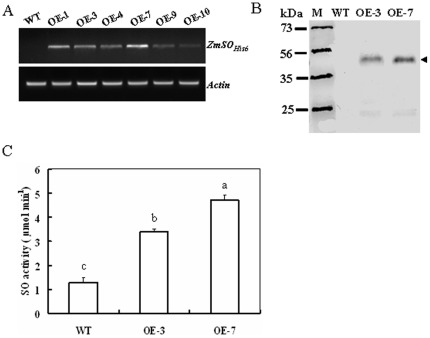
Expression levels and activity of ZmSO in SO-modified tobacco plants. (A) Transcription levels of the *ZmSO-His_6_* in wild-type tobacco plants and six homozygous over-expression (OE) lines (named OE-1, OE-3, OE-4, OE-7, OE-9, and OE-10). ZmSO transcripts detected by semi-quantitative RT-PCR were present in the OE lines, but not in the wild-type plants. Moreover, the ZmSO transcript level was highest in OE-7 among the six over-expression lines. (B) Western blot analysis of wild-type and OE lines (OE-3 and OE-7). Proteins (20 µg per lane) were fractionated by 12.5% SDS-PAGE and immunobloted with histidine tag-specific antibody. The protein level of *ZmSO-His_6_* was also relatively higher in OE-7 than that in OE-3. (C) Total SO activity in leaf extracts from wild-type and OE lines measured by kinetic assays. The total SO activity was significantly higher in the leaf extracts of OE-3 and OE-7 than in wild-type plants. Kinetics of SO activity was assayed using the ferricyanide reduction technique. The steady-state kinetics of ferricyanide reduction was followed spectrophotometrically at 420 nm using 10 g of protein extract.

To evaluate the *in vivo* physiological role of *ZmSO*, a CaMV 35 S promoter-driven binary expression construct harboring *ZmSO-His_6_* was developed and transformed into tobacco plants. To this end, six homozygous transgenic lines over-expressing *ZmSO* were developed, two of which (OE-3 and OE-7) were characterized in more detail. The transcription level of *ZmSO* was relatively high in OE-7 compared with that in OE-3 as was revealed by RT-PCR analysis (Fig. 2A). Consistent with this difference, the protein level of ZmSO was also relatively higher in OE-7 than in OE-3 when detected by Western blotting using a His-tag monoclonal antibody (Fig. 2B, arrowed). Compared with wild-type plants, total SO activities in the leaf extracts of both OE-3 and OE-7 were significantly elevated. In both OE lines, the SO activity was 1.5 and 2.5-fold higher than in wild-type plants, respectively (Fig. 2C).These results further confirm that overexpression of *ZmSO* results in increased SO activity *in planta*.

### Responses of *ZmSO* Expressing Tobacco Plants to Sulfite Stress

**Figure 3:**
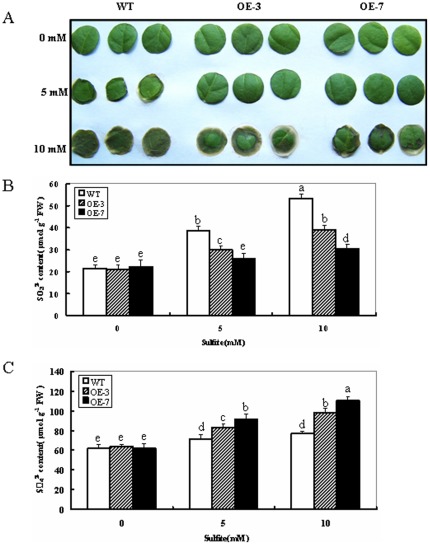
Responses of wild-type and ZmSO over-expression tobacco plants to toxic levels of sulfite. (A) Toxic effect of Na_2_SO_3_ (5 and 10 mM) on leaf discs from 8-week old wild-type and OE lines. After 16 h treatment, Leaf discs of wild-type plants showed higher chlorosis and damage than OE lines (OE-3 and OE-7). (B) Sulfite concentration in Na_2_SO_3_ treated leaves from wild-type and OE plants. (C) Sulfate concentration in Na_2_SO_3_ treated leaves from wild-type and OE plants. In both (B) and (C), leaf discs were separately sampled and washed three times with distilled water after exposure of the plants to 0, 5 and 10 mM Na_2_SO_3_ for 16 h. The leaves were then used for sulfite and sulfate contents analysis. Data are the means of three replicates (±SE). Means denoted by the same letter did not significantly differ at P<0.05. The data are from one of three different experiments that yielded essentially identical results.

The result above indicates that the transgenic tobacco lines (OE-3 and OE-7) have different amounts of SO protein and show differential activities. It was therefore of interest to examine the response of SO-modified tobacco plants to sulfite stress. Leaf discs of wild-type and transgenic plants were treated with 5 and 10 mM Na_2_SO_3_, respectively. After 16 h, the wild-type plants showed significantly higher chlorosis and necrosis than the OE lines (OE-3 and OE-7) in both 5 and 10 mM Na_2_SO_3_ treatments. This was clearly seen upon 10 mM Na_2_SO_3_ exposure (Fig. 3A). The results demonstrate that overexpression of *ZmSO* in transgenic tobacco plants significantly enhances tolerance to toxic sulfite.

To monitor *in planta* changes in the levels of ZmSO substrate and product, sulfite and sulfate contents from treated and control leaf discs were determined. In the wild-type plants, total sulfite content in the leaf discs increased by 78% for 5 mM Na_2_SO_3_ exposure and 147% for 10 mM Na_2_SO_3_ exposure, respectively. By contrast, sulfite levels in both OE lines only increased by 16 and 43% for 5 mM Na_2_SO_3_ exposure and 38 and 86% for 10 mM Na_2_SO_3_ exposure, respectively (Fig. 3B). For changes in the sulfate concentration, increases of 31 and 47% for 5 mM Na_2_SO_3_ exposure and 54 and 78% for 10 mM Na_2_SO_3_ exposure were detected in both OE lines, respectively. By contrast, the sulfate levels in wild-type plants resulted in increases of 15% for 5 mM Na_2_SO_3_ exposure and 24% for 10 mM Na_2_SO_3_ exposure, respectively (Fig. 3C).These results clearly indicate that OE lines with increased SO activity showed higher tolerance during sulfite stress due to their enhanced capacity to detoxify sulfite.

### H_2_O_2_ Accumulation in *ZmSO* Transgenic Plants During Sulfite Stress

**Figure 4:**
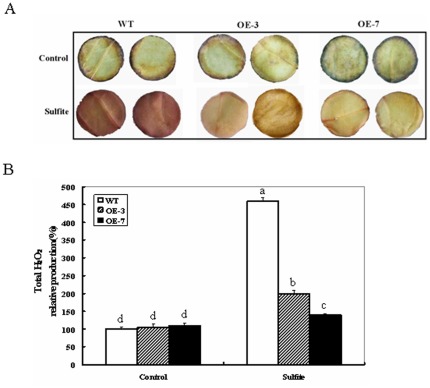
H_2_O_2_ accumulation in leaf discs of wild-type and ZmSO over-expressing plants in response to sulfite exposure. (A) H_2_O_2_ production in leaf discs of wild-type and OE plants treated with 5 mM Na_2_SO_3_ was visualized by staining with 3, 3′-diaminobenzidine (DAB). The leaf discs were treated for 16 h, and were subsequently stained with DAB as described in the experimental procedures. (B) Relative total H_2_O_2_ production was quantified in leaf discs from two independent experiments. Error bars indicate SE (n = 10). The lower case letters (a, b, c, d) indicate P<0.05 for the differences within treatment between ecotypes.

The phytotoxicity from sulfite may be associated with production of reactive oxygen species (ROS), which cause oxidative damage to proteins, DNA, and lipids in plant cells [26].In addition, the recombinant *Arabidopsis* SO protein expressed bacterially has been identified as a novel producer of H_2_O_2_ in biochemical assays [17]. This may be indicative of H_2_O_2_ accumulation during SO-dependent sulfite detoxification in wild-type and OE plants. Histochemical detection of H_2_O_2_ accumulation was performed with 3, 3′-diaminobenzidine (DAB) staining in sulfite-treated and control leaf discs from wild-type and OE plants. No significant differences in DAB staining intensity were observed between wild-type and OE lines after 16 h of distilled water-treated controls. When the discs were treated with sulfite, a significant increase was observed in DAB staining intensity (Fig. 4A). Surprisingly, the wild-type showed much higher DAB staining intensity than the OE lines (Fig. 4A). Quantitative determination of H_2_O_2_ accumulation further demonstrated that the wild-type increased by 3.6-fold in DAB staining intensity, while the OE lines only increased by less than 1-fold (90% for OE-3 and 26% for OE-7; Fig. 4B). These results further demonstrate that sulfite can induce ROS production, thus leads to oxidative stress. However, H_2_O_2_ accumulation in ZmSO transgenic lines is much less than in wild-type plants; indicating that ZmSO OE lines may have more efficient H_2_O_2_ scavenging than wild-type plants.

### Changes in Catalase Activity and *Cat* gene Expression in ZmSO OE Transgenic Plants During Sulfite Stress

**Figure 5:**
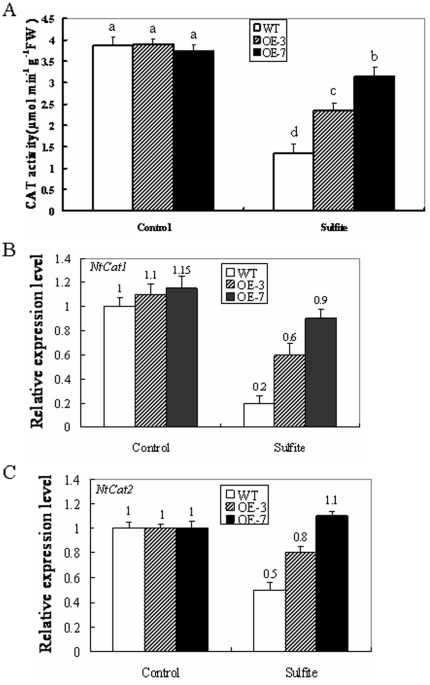
Effects of sulfite on catalase activity and gene expression in wild-type and ZmSO over-expression tobacco plants. (A) Effect of sulfite on total catalase (CAT) activity in wild-type and OE lines (OE-3 and OE-7). Leaf samples from wild-type and OE plants were harvested after 16 h of 5 mM Na_2_SO_3_ treatment and total CAT activities were analyzed. Values are means ± SE (n = 6). Means denoted by the same letter did not significantly differ at P<0.05. (B) Effect of sulfite on transcription levels of *Cat1* in wild-type and OE lines (OE-3 and OE-7). (C) Effect of sulfite on transcription levels of *Cat2* in wild-type and OE lines (OE-3 and OE-7). In both assays, leaf samples from wild-type and OE plants were harvested after 16 h of 5 mM Na_2_SO_3_ treatment and transcriptional expression of *Cat1* and *Cat2* was detected by qRT-PCR.

The differential H_2_O_2_ accumulation in the wild-type and OE lines may be caused by different catalase (CAT)-dependent H_2_O_2_ scavenging capability in peroxisomes. To check this hypothesis, CAT activities were examined in intact leaves from wild-type and OE lines during sulfite stress. After 16 h of 5 mM sulfite exposure, the wild-type displayed a 65% reduction in CAT activity whereas the two OE lines only showed reductions of 38% and 16% in CAT activity, respectively (Fig. 5A). These results demonstrated that CAT activities in the OE lines are reduced much less than in the wild-type. This indicates that higher levels of SO may play a role in protecting CAT from inhibition by excess sulfite. In return, high residual CAT activity can scavenge H_2_O_2_ accumulation efficiently in OE transgenic lines. As a result, high levels of SO and CAT contribute to less leaf damage in the OE lines during sulfite stress.

To further look at the changes in *CAT* gene expression levels during sulfite stress, transcripts of *Cat1* (accession number: U93244) and *Cat2* (accession number: U07627) from tobacco were monitored in the wild-type and OE leaves by qRT-PCR. After 16 h, the abundance of *Cat1* and *Cat2* transcripts displayed differential patterns between the wild-type and OE lines. Compared with controls, the transcript levels of *Cat1* were sharply down-regulated in the wild-type, whereas they were partially reduced in the OE lines. The wild-type showed an 80% reduction, while the two OE lines exhibited 45 and 20% reductions, respectively (Fig. 5B). By contrast, the transcription levels of *Cat2* were slightly up-regulated in the OE-7 line (10% increase). The OE-3 line showed small down-regulation of *Cat2* (20% reduction), but *Cat2* was still significantly down-regulated in the wild-type (50% reduction, Fig. 5C). The results further indicate that *ZmSO* plays a positive role in *CAT* gene transcription changes induced by toxic levels of sulfite.

## Discussion

In this study, we investigated biochemical properties and physiological roles of sulfite oxidase from maize using several complementary approaches. Our genetic evidence has strongly demonstrated that ZmSO is involved in sulfite detoxification and its over-expression may play a positive role in protecting peroxisomal CAT from inhibition by sulfite. To the best of our knowledge, this is the first sulfite oxidase gene from monocot plants to be functionally characterized.

### ZmSO has High Affinity Towards Sulfite

As expected, the recombinant ZmSO protein exhibited a sulfite-dependent SO activity when ferricyanide was employed as the electron acceptor (Fig. 1). No activity was found with cytochrome *c* as the electron acceptor (data not shown). Since the heme domain is missing within the protein, this may indicate that the natural electron acceptor *in vivo* is oxygen. This observation was also found in the SO from the model plant *Arabidopsis* and the bacterial *D.* *radiodurans* due to the absence of heme binding domains in the proteins [12], [27]. The *K_m_* for sulfite in the ferricyanide assay was determined to be 21.4 µM, which is much lower than the *K_m_* values for *Arabidopsis* (33.8 µM) [12], rat (33.0 µM) [14], or *Nicotiana tabacum* SO (51.4 µM), but in the same range of the values for human [28] and chicken [13] SO (17 and 19.1 µM, respectively). This suggests that ZmSO has higher affinity towards sulfite than *Arabidopsis* and tobacco SO proteins, whereas slightly lower affinity than those of human and chicken. The crystal structure of *Arabidopsis* SO has revealed that Arg374 is an important conserved residue for sulfite-binding [16]. Further studies will be interesting to identify the active site responsible for substrate binding and catalytic activity in the monocot ZmSO by site-directed mutagenesis.

### SO Protects Plants Against Sulfite Toxicity Predominantly via Sulfite Oxidation

Under normal conditions, the OE transgenic lines displayed no obvious phenotypic differences in appearance, flowering, and seed production compared with the wild-type plants when grown in soil (data not shown). When exposed to high concentrations of sulfite (5 and 10 mM Na_2_SO_3_), leaf discs of OE plants displayed less damage than wild-type plants (Fig. 3A). Sulfite and sulfate content determination revealed that smaller increases in the total sulfite and greater increases in sulfate were observed in both OE lines compared to wild-type plants regardless of 5 or 10 mM sulfite exposure (Fig. 3B,C). In other words, greater amounts of sulfite were transformed to nontoxic sulfate in the OE lines due to the increased SO activity levels. This is in good agreement with previous results from transgenic *Arabidopsis* with SO over-expression under SO_2_/sulfite exposure [19], [20].

In addition to the SO-dependent sulfite oxidation pathway in peroxisomes, a sulfite reduction pathway in chloroplasts can not be neglected. A recent study in *Arabidopsis* has showed that transcript and activity levels of sulfite reductase (SiR), a chloroplast-localized enzyme, which can convert sulfites into sulfides, were induced by sulfite, but its regulation was connected to SO activity levels during SO_2_/sulfite exposure [19]. This reinforces the view that SO-dependent sulfite oxidation is still the predominant pathway during sulfite detoxification in plants. Further work is needed to dissect the SO-dependent sulfite detoxification network using microarray analysis in SO-modified plants.

### SO may Play a Positive Role in Protecting CAT from Inhibition by Excess Sulfite

The recombinant *Arabidopsis* SO has been identified to be a novel producer of H_2_O_2_ as observed by *in vitro* biochemical assays [17]. Consistent with this phenomenon, H_2_O_2_ accumulation between wild-type and OE lines was observed during sulfite stress by histochemical staining and quantitative determination (Fig. 4A, B). Theoretically, greater amounts of H_2_O_2_ should be produced in the OE lines than in the wild-type during sulfite detoxification, but much less H_2_O_2_ accumulation was observed in the OE lines (Fig. 4). CAT is essential for the removal of H_2_O_2_ produced in the peroxisomes [29]. The activity levels of CAT in both wild-type and OE lines were down-regulated by sulfite stress, but much less reductions of CAT levels were detected in the OE lines than in the wild-type plants (Fig. 5). Therefore, less H_2_O_2_ accumulation in the OE lines may attribute to more residual CAT-dependent H_2_O_2_ scavenging during sulfite stress.

Hansch et al (2007) proposed that SO could play a role in protecting catalase from sulfite damage [30]. However, no direct genetic evidence was available to support this view. In this study, we provided genetic evidence that SO plays a positive role in protecting CAT from inhibition by excess sulfite using the SO-OE tobacco plants. Further genetic work is needed to validate the interaction between SO and CAT at the molecular level using suitable SO-knockout lines during sulfite stress *in planta*.

In summary, these data clearly demonstrate that the monocot *ZmSO* can detoxify excess sulfite through sulfite oxidation and protect peroxisomal CAT from inhibition during sulfite detoxification in plants.This study will facilitate our understanding of the biological roles of SO in higher plants and accelerate genetic improvement of crop plants that have tolerance to environmental pollutants (SO_2_ and acid rain, etc).

## Materials and Methods

### Plant Materials and Growth Conditions

Tobacco plants (*Nicotiana tabacum* cv. Xanthi) were grown as aseptic shoot cultures on 1/2 MS medium for gene transformation, and transgenic plants were grown in a growth room at approximately 26°C, a photoperiod of 16 h/8 h (day/night) and light intensity of 200 µmol m^−2^ s^−1^.

### Expression, Purification and Kinetic Analysis of Recombinant ZmSO

The *ZmSO* cDNA was amplified as a *Kpn*I/*Xho*I (underlined) fragment by PCR using primers SO-F1∶5′-AGAGGTACCATGCCCGGGCTCACGGC-3′ and SO-R1∶5′-ACA CTCGAGTCACAGCTTAGATCTTTC-3′. The fragment was cloned into the vector pET-30a (Novagen, USA), resulting in the bacterial expression construct pET-ZmSO. The expression construct was transformed into *E. coli* BL21 (DE3) pLysS cells and would produce a recombinant ZmSO containing a 6×histidine tag at its N-terminus. The cells harboring pET-ZmSO were induced with 0.1 mM isopropylthio-β-galactoside (IPTG). The over-expressed ZmSO was purified using nickel chelate affinity purification kit according to the supplier’s instructions (Sangon Co., Shanghai, China). The resulting peak elution fractions were characterized by 12.5% SDS-PAGE and Western blotting using the anti-histidine monoclonal antibody (Bio Basic Inc, Canada). The protein concentration was determined as described by Bradford [31]. Kinetic measurements of SO activity were determined as mentioned above using a saturating concentration of ferricyanide (400 µM) and various concentrations of sulfite between 2.5 and 400 µM [12]. The *K_m_* and *V_max_* values were estimated from the Lineweaver-Burk plots by plotting reaction rates versus increasing concentrations of substrate. The assays were each repeated at least three times, and the kinetic constants were all determined using the data of three independent assays.

### Construction of Plant Expression Vectors and Development of Transgenic Tobacco Lines

The full-length *ZmSO* cDNA was amplified and introduced into the pART7 plasmid using primers SO-F2∶5′-AGAGAATTCATGCCCGGGCTCACGGCAC-3′ containing *Eco*RI restriction site (underlined) and SO-R2∶5′-ACAGGATCCTCAGTGGTGGTGG TGGTGGTGCAGCTTAGATCTTTCAAC containing *Bam*HI restriction site (underlined). The reverse primer used for this amplification contained the coding sequence for the 6×histidine peptide tag. This gave rise to the fusion gene *ZmSO-His_6_*, which was subsequently inserted downstream of the 35 S promoter in the plasmid vector pART7. The resulting expression cassette containing the 35 S promoter and *ZmSO-His_6_* was cut and inserted into the binary vector pART27 [32], producing the transformation construct pART27-35S:: ZmSO-His_6_. The binary construct was then introduced into *Agrobacterium tumefaciens* strain LBA4404 for tobacco transformation.

Leaf discs of *N. tabacum* cv Xanthi were used for gene transformation via *A. tumefaciens*. The regenerated explants were cultivated and transplanted to fresh MS medium with corresponding antibiotic resistance every 2 weeks. Excised shoots were then transferred to the hormone-free MS medium supplemented with 50 mg l^−1^ kanamycin for root induction [33]. These kanamycin-resistant plantlets were confirmed by PCR with primers SO-F3∶5′-TGCCCGGGCTCACGGCAC-3′ and SO-R3∶5′- CAGTGGTGGTGGTGGTGGTGC-3′ (the reverse primer SO-R3 only contains coding sequence of the 6×histidine peptide tag) for *ZmSO* transgenic tobacco. The PCR-positive plantlets were transplanted into soil for growing in the growth room. The transgenic tobacco progenies were selected using 1/2 MS plate containing 50 mg l^−1^ kanamycin and maintained growth to set seeds in soil until T_2_ generation. Six independent homozygous ZmSO over-expression (OE) transgenic lines (named OE-1, OE-3, OE-4, OE-7, OE-9, and OE-10) were developed. The expression of the transgene in the six lines was evaluated by semi-quantitative RT-PCR with SO-F3 and SO-R3.The cDNA contents of different reverse transcription reactions were normalized by amplifying *Actin* transcripts using a pair of primers Actin-F: 5′-GGCAGCTCGTAGC TCTTCTC-3′ and Actin-R: 5′-AACAGGGAGAAGATGACCC A-3′, which produces an 874-bp product. Based on the results of the semi-quantitative RT-PCR experiments, two representative OE lines (OE-3 and OE-7) were selected for further use. The expression levels of ZmSO protein in the two lines were investigated using Western blot analysis with the anti-histidine monoclonal antibody.

### Treatment of Sulfite for Tobacco Leaf Discs

The fifth fully expanded leaves from 8-week-old wild-type and transgenic plants were used for sulfite stress assay. Leaf discs (15 mm Φ) were placed in Petri dishes (150 mm Φ) on a filter paper moistened with either 5 mM or 10 mM Na_2_SO_3_ solution (3 ml) for the specified times under constant illumination (200 µmol m^−2^ s^−1^), and then were photographed and rinsed with de-ionized water for sulfite and sulfate content determination and enzyme activity analyses.

### Histochemical Detection and Quantitative Determination of H_2_O_2_ Production

The histochemical detection of H_2_O_2_ from sulfite-treated leaf discs with 3, 3′- diaminobenzidine (DAB) was performed according to the method of Rea et al. [34]. After staining, the leaf discs were rinsed in 80% (v/v) ethanol for 10 min at 70°C, mounted in lactic acid:phenol:water (1∶1∶1, v/v/v), and photographed directly using a SONY digital camera (SONY DSC-F828). H_2_O_2_ content was assayed according to the method of Ferguson et al. [35].

### Catalase Activity and gene Expression

Frozen leaf samples (0.5 g) were crushed into powder in liquid N_2_. Crude proteins were extracted and used for the determination of catalase (CAT) activity [36]. CAT gene expression in sulfite-treated tobacco plants was conducted by quantitative real-time PCR (qRT-PCR). Gene sequences from tobacco were obtained as described by Pasqualini et al. [37]. Gene-specific primers for tobacco are: *NtActin* Act-F: 5′-GTGCTGAGCG TTTCCGTTGT-3′ and Act-R: 5′-CTGCAGCTTCCA TTCCA ATCA-3′; *NtCat1* Cat1-F: 5′-ACAAGTACCGTCCGTCAAGTGC-3′ and Cat1-R: 5′-TCAATGTGAATGTGTGG ACACC-3′; *NtCat2* Cat2-F: 5′-TGTGGTGTCAAGTGCATGTCG-3′ and Cat2-R: 5′-TGG GTACTGTTCAGCATGACG-3′. Criteria for designing primers were a primer size between 20 and 25, an optimal T_m_ at 60 °C, and a product size ranging from 180 bp to 250 bp. Amplification of *Actin* (accession numbers: AF15640) was used as internal control for tobacco and qRT-PCR assays were performed on an IQ5 light cycler (Bio-Rad) using SYBR Premix ExTaq II (Takara, Japan) with gene-specific primers above. For the entire qRT-PCR assay, three technical replicates were performed for each experiment and the expression of each gene was investigated in three biological replicates.

### Western Blot Analysis

Twenty micrograms of protein were electrophoretically separated on 12.5% SDS-PAGE and transferred onto a nitrocellulose membrane. The membranes were blocked and thereafter blotted with a commercial His-tag monoclonal antibody for 3 h at a 1∶2000 dilution. After extensive washing, the bound primary antibody was detected with a horseradish peroxidase-conjugated goat anti-mouse IgG secondary antibody using the 3, 3′-diaminobenzidine (DAB) development kit according to the manufacturer’s protocol (Bio Basic Inc, Canada).

### Biochemical Assays of SO Activity

SO activity was determined by the reduction of ferricyanide at 420 nm in a reaction mixture containing 10 µg soluble protein, 0.395 mM ferricyanide, and 0.4 mM sodium sulfite in 2 ml of 20 mM Tris-HCl buffer (pH 8.0). The reaction mixture without sodium sulfite served as a control, and one unit of SO activity was defined as the conversion of 1 mmol sulfite into sulfate min^−1^ [12], [21].

### Determination of Sulfite and Sulfate Contents

Sulfite and sulfate contents from sulfite-treated leaf discs were measured using an ion exchange chromatography system as described previously [17], [21]. For each experiment, three replicates were conducted for each test sample and the experiment was repeated three times.
